# Natural human knockouts and the era of genotype to phenotype

**DOI:** 10.1186/s13073-015-0173-z

**Published:** 2015-05-29

**Authors:** Fowzan S Alkuraya

**Affiliations:** Department of Genetics, King Faisal Specialist Hospital and Research Center, Riyadh, Saudi Arabia; Department of Anatomy and Cell Biology, College of Medicine, Alfaisal University, Riyadh, Saudi Arabia

## Abstract

Complete loss of gene function in humans by naturally occurring biallelic loss-of-function mutations (human knockout) is not a new concept. However, the recent identification of human knockouts along the entire spectrum of health and disease by next-generation sequencing promises to unlock their full potential to accelerate the medical and functional annotation of the human genome.

Next-generation sequencing of nucleic acids has done more than amplify the throughput of sequencing by orders of magnitude; it has eliminated a bottleneck that shaped the way in which human genetics and, more specifically, medical genetics was practiced. Free of the constraint of having to develop a hypothesis to obtain relevant sequence information, human geneticists are now reveling in the flood of sequence data that is available to them with no prerequisites other than sample availability. The implications of this paradigm shift are far reaching and have yet to be fully captured. One such implication is the rapidly growing trend of genotype-to-phenotype approaches in which hypotheses about the phenotype are generated after analyzing the sequence data rather than before. No class of variants lends itself to this new approach more readily than loss-of-function (LOF) variants, which ‘knock out’ the involved gene such that their biallelic presence essentially makes the individual into a human knockout for that gene. If there are pathological consequences to loss of a gene’s function, human knockouts for that gene offer the best opportunity to study such consequences.

## Human knockouts for known disease genes

The clinical consequences of mutations in established disease genes usually depend on the disease’s molecular pathogenesis (dominant negative, dominant gain of function, loss of function, and so on) but even then, the results of complete knockouts can be highly surprising. For example, *BRCA2* and *APC* are well established dominant disease genes for breast/ovarian cancer and colon cancer, respectively. However, human knockouts for *BRCA2* (primordial dwarfism) and *APC* (severe limb malformation) have astonishingly different phenotypes from those of the established dominant phenotype in haploinsufficient individuals [1, 2]. Even for known recessive disease genes, human knockouts can have a dramatic phenotype that bears little or no resemblance to the well established recessive disease; for example, the *NEB* gene, which encodes a large cytoskeletal protein, causes embryonic lethality in human knockouts, whereas in those with less severe biallelic mutations it only causes myopathy [3]. On the other hand, healthy people who have knockouts in genes previously thought to cause disease in a recessive manner can raise doubts about the originally proposed disease links [4]. One particularly exciting potential of human knockouts is to unmask the true disease potential of genes that have previously only been associated with rather than linked to human disease. For example, *DNASE1L3* is associated with systemic lupus erythematosus, a complex multifactorial disorder, but its complete knockout directly causes this disease in a Mendelian recessive manner [5]. Human knockout events in known disease genes can also serve as a bridge between Mendelian and complex phenotypes in other ways. For example, we now know from large-scale sequencing projects that knockout events in genes known to exert Mendelian phenotypes related to glucose and lipid homeostasis also have an important role in the well known risk variation between individuals for diabetes mellitus and cardiovascular disease [5–7].

## Human knockouts for genes not known to cause diseases

Traditionally, the identification of a human knockout for a novel gene represented compelling evidence for establishing novel disease-gene links. Such causal links were derived in the context of a positional mapping strategy that attaches a compelling probabilistic value to the locus. However, recent studies have unveiled a previously unrecognized level of human knockout events. MacArthur *et al.* [8] found that healthy people on average have about 15 complete knockout events (not counting splicing mutations that may or may not lead to complete knockout) [8]. In the highly consanguineous Saudi Arabian population, we found that in the offspring of first cousin parents there were on average 23 complete knockout events, and this larger number was mainly driven by the high percentage of autozygosity (identity by descent) [4]. Similarly, the recent study by Stefansson and colleagues showed that the Icelandic population, with its strong founder effect, has a large burden of complete knockout events [9]. These studies clearly show that a knockout event in a novel gene does not necessarily mean that this gene causes an individual’s phenotype. More importantly, these studies truly usher in an era of genotype-to-phenotype approaches with all the exciting possibilities that follow.

## Reverse phenotyping from large-scale human knockout studies

Cohorts sequenced to date in search of human knockouts have given different estimates of the number of knockout events per person depending on whether they were enriched for complete (biallelic) knockout events. The study by Stefansson and colleagues [9] is particularly noteworthy not only because of the size of the sequenced cohort (2,636) or its enrichment for biallelic knockout events (due to the founder effect of the Icelandic population) but also because the LOF alleles were imputed by high-density genotyping such that the cohort size screened for these events was effectively increased to >100,000 [9]. This is by far the largest study to date on human knockouts and provides a valuable resource in the discovery of the role of complete human knockouts in general populations. Although the study was performed on a genetic isolate, some of its results seem to be generalizable; for example, the predilection of knockout events to affect olfactory genes was similarly observed by MacArthur *et al.* [8] and Alsalem *et al.* [4]. As the study cohort was mixed, their impressive list of about 5,000 genes with biallelic LOF [9] should not be interpreted as a list of genes that do not cause severe Mendelian diseases in humans. For instance, they proposed that their knockout subjects for *LRIG3* and *OTOP1* are candidates for auditory evaluation to assess for hearing loss given the established role for these genes in ear development [9]. On the other hand, the cohorts studied by MacArthur *et al.* [8] and Alsalem *et al.* [4] were selected such that severe Mendelian diseases (or their casual variants) were excluded. The resulting partially overlapping lists of 221 and 169 genes, respectively (the former excluding splicing variants), were therefore significantly depleted for severe Mendelian phenotypes. Consequently, these knockout events represent a rich resource to generate hypotheses about the apparent tolerance of the human genome to these knockout events.

One of the most intriguing, testable hypotheses is that this tolerance is context dependent. The context could be genetic or environmental. For example, there are numerous single knockouts in model organisms that appear completely normal but a phenotype is observed in double knockouts [5]. A second knockout event is not necessary, however, because milder genetic modifiers (referred to as genetic background in mouse models) can also be important in determining the phenotypic expression of the human knockout events as they do in knockout animal models. Environmental factors may also be important determinants; for example, *FUT2* knockout may lead to clinically consequential B12 deficiency only in nutrition-deficiency states [4]. We have also observed knockout events in several immune-related genes and it is possible that these will only manifest phenotypically under certain microbial exposures [4]. Knockout events may have a role in human phenotypic diversity in less subtle ways [5]. For example, knockout of olfactory and keratin genes may influence flavor preferences and hair texture, respectively. These are just two of the many phenotypes that are not typically assessed upon enrolling ‘healthy’ individuals in these sequencing studies, so it is inevitable that reverse phenotyping will become commonplace as we move from the discovery of knockout events in genes to evaluating their potential phenotypic consequences, taking a lead from their known or predicted biological roles [5].

## A field in its infancy

Human knockout research has already demonstrated a great potential despite being in its very early stages. What is clear is that large-scale sequencing studies from cohorts enriched for autozygosity will provide the necessary statistical power to capture viable human knockout events. When such large datasets are available, interesting questions can be asked and answered in terms of what a particular gene knockout event can cause and in what context. *PCSK9*, knockouts of which protect individuals from cholesterol-driven cardiovascular diseases, has become the poster child for the immense pharmaceutical potential of human knockout research. Drugs that block PCSK9 (mimicking the naturally occurring human knockouts) are among the most promising lipid lowering agents ever since statin class drugs were introduced, and they were inspired solely by the cardiovascular benefits enjoyed by humans who have a knockout of this gene [5]. It is inevitable that such research will uncover knockouts that could be tapped for similarly successful therapeutic opportunities [5]. The appreciation of the influence of knockout events on the individual risk for complex diseases is also very likely to see significant growth in the near future [10, 11].

The stereotype of human knockouts as individuals with esoteric severe diseases will quickly become a thing of the past. Human geneticists are now bracing themselves for human knockouts to be very much part of the genetic individuality that drives personalized medicine, and it is expected that the wider medical community will follow.
